# Nanomaterials for the Diagnosis and Treatment of Urinary Tract Infections

**DOI:** 10.3390/nano11020546

**Published:** 2021-02-22

**Authors:** Maimoona Qindeel, Mahmood Barani, Abbas Rahdar, Rabia Arshad, Magali Cucchiarini

**Affiliations:** 1 Department of Pharmacy, Quaid-i-Azam University, Islamabad 45320, Pakistan; mqindeel81@gmail.com (M.Q.); rabia.arshad@bs.qau.edu.pk (R.A.); 2 Department of Chemistry, Shahid Bahonar University of Kerman, Kerman 76169-14111, Iran; mahmoodbarani7@gmail.com; 3 Department of Physics, Faculty of Science, University of Zabol, Zabol 538-98615, Iran; 4 Center of Experimental Orthopaedics, Saarland University Medical Center, Kirrbergerstr. Bldg. 37, D-66421 Homburg, Germany

**Keywords:** urinary tract infections, multidrug resistance, surface-tailored nanomedicines, diagnosis, therapy

## Abstract

The diagnosis and treatment of urinary tract infections (UTIs) remain challenging due to the lack of convenient assessment techniques and to the resistance to conventional antimicrobial therapy, showing the need for novel approaches to address such problems. In this regard, nanotechnology has a strong potential for both the diagnosis and therapy of UTIs via controlled delivery of antimicrobials upon stable, effective and sustained drug release. On one side, nanoscience allowed the production of various nanomaterial-based evaluation tools as precise, effective, and rapid procedures for the identification of UTIs. On the other side, nanotechnology brought tremendous breakthroughs for the treatment of UTIs based on the use of metallic nanoparticles (NPs) for instance, owing to the antimicrobial properties of metals, or of surface-tailored nanocarriers, allowing to overcome multidrug-resistance and prevent biofilm formation via targeted drug delivery to desired sites of action and preventing the development of cytotoxic processes in healthy cells. The goal of the current study is therefore to present the newest developments for the diagnosis and treatment of UTIs based on nanotechnology procedures in relation to the currently available techniques.

## 1. Introduction

The urinary system is the most vital organ system, responsible for the drainage of urine and for the maintenance of homeostasis [1] via regulation of the blood volume, pressure, and pH and of metabolites and electrolytes [2]. The urinary system comprises the kidneys removing wastes and water from the blood in the form of urine [3] that is carried towards the bladder where it gets stored before leaving the body via the urethra [4].

Infections in any part of the urinary system lead to the development of urinary tract infections (UTIs) [4,5]. UTIs are considered as one of the most common infections caused by bacteria, with 150 million people annually affected worldwide [6]. UTIs can be characterized as complicated or uncomplicated [7]. Complicated UTIs are mostly comprised of infections associated with compromised urinary tract and immune system [7]. Common diseases associated with complicated UTIs include neurological impairment-based urinary obstruction, urinary retention, and immunosuppression, renal transplantation, renal failure, pregnancy, or foreign bodies such as biofilms, calculi, catheters, and other devices [8]. Catheter-associated UTIs (CAUTIs) are linked to increased morbidity and mortality and are the most common origin of secondary bloodstream infections [9,10]. Non-complicated UTIs, the most prevalent forms of UTIs, are not based on any neurological, structural, nor physiological defects of the urinary tract [11], characteristically affecting women, children, and old patients. They are divided into upper tract UTIs in the nephron (pyelonephritis) and lower tract UTIs (cystitis). The most prominent etiological risk factors for uncomplicated UTIs are chronic UTIs, sexually transmitted UTIs, vaginal infections, diabetes, obesity, and genetics [12,13]. UTIs are mostly caused by both Gram-positive and Gram-negative bacteria and by some fungi. Uropathogenic *Escherichia coli* (UPEC) is the only common causative agent in the case of both complicated and uncomplicated UTIs [14,15]. Peculiar microbes involved in the pathogenesis of complicated UTIs also include *Enterococcus* spp., *Klebsiella pneumonia*, *Staphylococcus aureus*, *Pseudomonas aeruginosa*, and *Candida* spp. [16]. Uncomplicated UTI pathogenesis also steeps progressively due to pathogenic *Klebsiella pneumoniae*, *Staphylococcus saprophyticus*, *Enterococcus faecalis*, group B *Streptococcus* (GBS), *Proteus mirabilis*, *Pseudomonas aeruginosa*, *Staphylococcus aureus*, and *Candida* spp. [17]. The basic mechanism involved in the pathogenesis of UTI is the adherence of specific virulence factors residing on bacteria in the urethra followed by colonization and further movement of the pathogen in the bladder using its appendages (flagella and pili) [18]. After attachment of the pathogens in the bladder, complexed host–pathogen interactions then occur, bringing the disease to advance stages. UPEC also develops biofilms-based intracellular bacterial communities (IBCs) as a protection against host immune responses and to overcome bacterial resistance [19]. UTIs result in compromised socioeconomical factors and health, affecting the quality life of affected individuals [20]. Currently, medications used for UTIs include trimethoprim sulfamethoxazole, ciprofloxacin, ampicillin [21], and combined antibiotic therapy to address the multidrug-resistance (MDR) features of virulent bacteria [22]. Vaccines targeting bacterial adhesions and bacterial toxins are also available [23,24]. Nevertheless, all these modalities are associated with an increased economic burden, non-patient compliance, and need for targeted delivery, and most importantly with the involvement of virulent factors in bacterial genes causing MDR, with the emergence of even more resistant genes, making the treatment of UTIs particularly arduous [25].

The diagnosis of UTIs initially requires a complete disease and medication history, followed by the physical examination of the pelvic region and the assessment of uropathogens in urine samples using various diagnostic approaches [26]. Laboratory diagnostic tests for UTIs include urinalysis, with an aim to reduce the dose of recommended antibiotics. Microscopic analysis of urine is also performed to diagnose the presence of certain microbes [27]. Culturing method is another standard diagnostic test to detect bacteria [28]. Moreover, the isothermal calorimetry technique is based on the evaluation of the metabolic rates of living microorganisms and microbes. Other conventional and common diagnostic techniques for UTIs include spectroscopic analysis such as Raman spectroscopy, Fourier transform infrared spectroscopy (FTIR), and UV spectroscopy [29,30]. Immunological diagnostic techniques like latex agglutination, enzymatic and coagulation assays are also useful in detecting antibodies and antigens of bacteria in the samples of UTI patients [26]. The diagnosis of UTIs is challenging owing to the vast existence of gaps accompanying over-testing, over-diagnosis, over-treatment, non-specificity, and heterogeneous nature of uropathogens [26,31].

Nanomaterial drug delivery is advantageous compared with conventional therapy because of enhanced specificity in targeting cells, altered pharmacokinetics as well as increased bioavailability, controlled drug release, increased solubilization of hydrophobic drugs, synergistic combinatorial chemistry, and enhancement in drug delivery [32,33,34,35,36]. Nanomaterials can exert their innate antimicrobial action, and synthesizing NPs while conjugating them to antimicrobials may lead to synergistic antibacterial actions, with cost-effectiveness and higher stability for a longer period [37]. Antimicrobial NPs for UTIs are mostly based on metals (gold - Au, silver - Ag, titanium NPs) and on metal-based oxides (zinc oxide-ZnO-NPs) [37,38,39,40,41,42,43,44]. All metal and metal-based oxide NP damage the pathogenic microorganisms by various mechanisms of photothermolysis, generating reacting oxygen species (ROS), damaging the cell wall and cell components of pathogens, and interfering with normal enzymatic activities and DNA synthesis [45].

The use of metallic NPs has several limitations such as the presence of hazardous wastes, the difficulty in scale-up production, instability, aggregation, and the use of expensive organometallic NPs [46,47]. To overcome these issues, green NP synthesis is being developed based on the use of natural extracts by exploring biological components, essential phytochemicals like flavonoids, terpenoids, alkaloids, phenols, and aldehydes as reducing agents and solvent systems. Moreover, to achieve surface functionalization and receptor-targeted action, the NP surface may be engineered with various biocompatible ligands, resulting in environmental remediation in terms of removing pollutant dyes and heavy metals [48,49,50].

Several nanoparticle-based antimicrobials are used against UTIs such as surfactant-based vesicles, nanoemulsions, and polymeric NPs like chitosan NPs [51,52]. Chitosan NPs have the ability to bind with negatively charged bacterial membranes and to increase their permeability. Carbon-based NPs and nitric oxide (NO)-releasing NPs can generate reactive nitrogen species (RNS) that also damage the bacterial membrane [53,54].

To overcome the limitations of conventional diagnostic methods for UTIs, a significant number of nanotechnology-based bioassays are highly effective [55,56,57,58,59,60]. One of these important techniques is optical imaging based on differences in contrast agents between different tissues. The Au nanorods are specific contrast agents, aiming at higher absorbance and scattering light and playing a promising role in damaging bacteria [61]. Similarly, magnetic NPs are required for precise quantification and targeting of pathogenic microbes. Probes based on Au NPs are highly effective and sensitive in detecting microbes [62,63,64,65,66,67]. Au nanowires arrays get attached with *Escherichia coli* antibodies to detect the peculiarity of UTIs [68]. MDR bacteria can be distinguished from non-resistant bacteria using Raman spectroscopic fingerprints. Moreover, the metabolic activity and antimicrobial susceptibility of pathogens can be evaluated via Con-A-conjugated supermagnetic iron oxide (SPIO) nanosensors [69]. Keeping in mind all the efforts related to the synthesis of novel nanomaterials and the investigation of their potential in bioapplications [39,40,70,71,72], we next review the nanomaterials applied to the diagnosis and treatment of UTIs.

## 2. Current Approaches for UTI Diagnosis and Biomarkers

UTIs are the most prevalent infections in persons of any age but especially in elderly people [73,74,75]. Owing to the high incidence and recurrence of UTIs and to the worldwide rise in antibiotic-resistant bacteria, both the diagnosis and treatment of lower and upper UTIs are becoming more difficult in clinical settings. There are also inexplicable or asymptomatic clinical effects of UTIs. Due to the possibility of septicemia and long-term effects, an early diagnosis of UTIs is critical [76,77,78,79].

The diagnosis of UTIs is currently focusing on the evidence of clinical symptoms combined with findings of the nitrite strip test (presence of urinary bacteria) and with an estimation of the number of white blood cells in urine [80,81], as UTI diagnosis in urine culture is time-consuming and costly [82,83]. Considerable efforts have been lately directed to evidence novel UTI biomarkers for diagnosis purposes. Several promising serum and urine biomarkers of UTIs like leukocyte esterase, heparin-binding protein, C-reactive protein (CRP), procalcitonin, lactoferrin, interleukins, C-reactive protein, elastase alpha (1)-proteinase inhibitor, secretory immunoglobulin A, heparin-binding protein, α-1 microglobulin (α1Mg), xanthine oxidase, soluble triggering receptor expressed on myeloid cells-1, myeloperoxidase, and tetrazolium nitroblue test (TNB) gained much attention in recent years [81]. The challenging problem is to quantitatively estimate these biomarkers when present at low concentrations. Specific and accurate identification of infectious pathogens in the healthcare industry is the key feature to improved patient care, select empirical treatment, and avoid the transmission of infections. Therefore, various diagnosis methods have been suggested for UTIs. Some may have slower responses, others prolonged response time, and some may have less precision while some may be too costly [84,85,86,87]. Nanotechnology may have the potential to overcome these issues and to increase the precision of the existing approaches [88,89].

## 3. Diagnosis of UTIs by Nanotechnology

**Figure 1:**
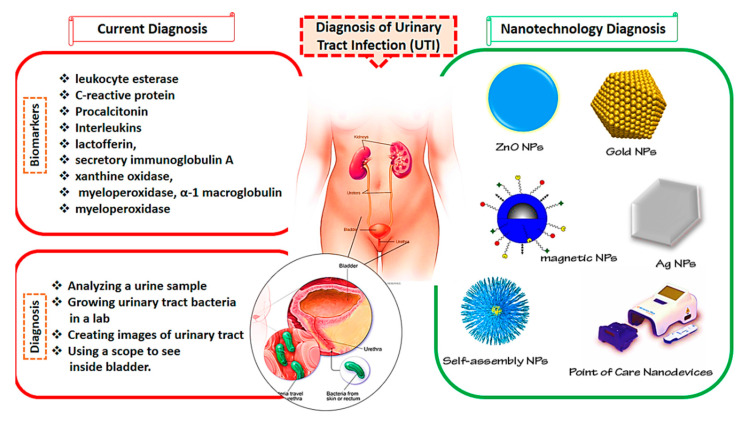
Current methods and nanotechnology approaches for the diagnosis of urinary tract infections (UTIs).

UTIs are among the most common bacterial infections, affecting about 50% of the population at least once in their lives. If UTIs are left undiagnosed and not properly handled, they may have deadly consequences. Additionally, finding a rapid, early, and reliable approach for the identification of uropathogens can be valuable in the clinical phase. Nanoscience is a highly developed area with tremendous scientific interest due to its numerous potential applications in the biomedical area [90]. To overcome the problems caused by traditional diagnosis approaches, nanomedicine can be combined with these methods for better management of UTIs. Nanostructures are more effective in preventing toxicity and resistance and reducing the costs of more traditional methods [91,92,93,94]. The current UTI diagnosis methods and nanotechnological approaches are presented in Figure 1.

As mentioned above, the existing approaches of urinalysis are complex and time-consuming and they lack both accuracy and precision [95]. Due to their fast response time and improved precision and accuracy, photoluminescence (PL)-based biosensors attracted more attention in recent years [95,96,97,98]. Concerning this, Vasudevan et al. [95] developed a nanosensor based on cysteamine-attached ZnO NPs (ZnO-Cys) for the identification of N-acyl-homoserine lactones (AHLs) Gram-negative bacteria. AHLs are responsible for the activation of pathogenicity in humans. ZnO NPs were prepared using a microwave-assisted approach with good sensitivity (97%) and a linear detection area of 10–120 nM in artificial urine media. The nanosensor was certified using the AHLs created by *Pseudomonas aeruginosa* (MCC3101) in real-time investigation, supporting their overall sensitivity and specificity.

In an attempt to early diagnose UTIs, a portable bacteria-grabbing nanochip based on surface-enhanced Raman scattering (SERS) was generated by Yang et al. [99] to detect three species of uropathogens (*Proteus mirabilis* PRM1, *Escherichia coli* CFT 073, *Pseudomonas aeruginosa* PAO1) directly from the urine sample medium. The chip was first functionalized with NH_3_^+^ groups for improved electrostatic adsorption of bacteria with a negative charge. Once the bacteria were captured by the chip, Ag NPs were used to achieve Raman fingerprint spectra of the bacteria. The SERS-based chip detected three species of UTI bacteria at low concentration (10^5^ cells/mL) by discriminant analysis (chemometric approach). Additionally, without any sample pre-treatment, the nanochip provided the fingerprint information of the bacteria directly from artificial urine and Luria–Bertani (LB) culture medium.

In another study, Alhogail et al. [100] created a rapid, adaptive, and precise colorimetric nanosensor based on magnetic NPs (MNPs) for the identification of *Pseudomonas aeruginosa* in vivo. The nanoplatform was based on a particular protease substratum assessment of *Pseudomonas aeruginosa* proteolytic activity. The substrate was covalently attached to MNPs on its N-terminus and connected to an Au sensor surface on its C-terminus. Generally, the Au nanosensor appears black to the naked eye due to coating with MNPs but at proteolysis, the peptide-MNP moieties are cleaved and absorbed by an external magnet. Thereafter, the golden color of the sensor surface becomes observable by the naked eye. In vitro, the biosensor identified the presence of *Pseudomonas aeruginosa* with 102 colony-forming units (cfu)/mL in less than 1 min while was capable of successfully detecting *Pseudomonas aeruginosa* in samples from patients.

In resource-limited environments, the point-of-care identification of pathogens in biological samples needs to be rapid, simple, cost-effective, compact, precise. Michael et al. [101] generated a custom-made fidget spinner based on Au NPs that rapidly concentrate the pathogens more than 100 times in 1 mL samples of undiluted urine for on-device colorimetric identification of bacterial loads and pathogen detection. The system allowed for the on-site, naked-eye identification of infection in urine samples from 39 patients suspected of having UTIs within 50 min. The authors also demonstrated that the system could be applied in 30 clinical samples of UTIs to conduct an antimicrobial susceptibility experiment within 120 min for the antimicrobial drugs ciprofloxacin and cefazolin. This fidget spinner may be used as an affordable portable system for the rapid concentration and identification of pathogens in urine samples in low-resource settings.

A mobile origami sensor was developed by Adrover-Jaume et al. [102] capable of detecting UTIs caused by *Escherichia coli* in less than 7 min. A single piece of paper containing antibody-decorated Au NPs was made from an origami nanosensor. If a sample of urine would contain *Escherichia coli*, the biosensors would produce colored spots on the paper strip that may be quantified by a mobile app by pixel measurements in real-time. The examinations were highly precise and did not crossreact with other uropathogens. In addition, when tested using a panel of 57 urine samples from patients, the biosensors only yielded one false-negative test, demonstrating their strong specificity and sensitivity. This result, along with a quick assessment time and smartphone-based identification, makes such biosensors a useful system to accurately identify UTIs.

Due to their excellent efficiency, low cost, and capacity to identify a broad range of target molecules such as nucleic acids and protein biomarkers, electrochemical nanosensors are well adapted for urinary diagnostics [103,104]. For a rapid measurement of the UTI lactoferrin biomarker, Pan et al. [105] created an electrochemical immunosensor (nanoarray of self-assembled monolayer alkanethiolate) from infected serum samples. Lactoferrin is a biomarker of pyuria (white blood cell involvement in urine), an essential UTI symptom [106]. The limit of detection (LOD) of the study was 145 pg/mL and the simultaneous identification of bacterial nucleic acid (16S rRNA) and of host immune response-associated protein (lactoferrin) on a single sensor array showed multichannel detection of uropathogens and lactoferrin. These findings provided the first interconnected nanoplatform for both quantitative pathogen detection and host immune response assessment.

There are some drawbacks to the commercially available colorimetric urine dipstic for early UTI detection. Identification and quantification of urinary leukocyte esterase (LE) remain unclear for the prediction of UTIs [107]. To solve this problem, Ho et al. [108] proposed a paper-based analytical device (PAD) to detect LE (LE-PAD) as a proof-of-concept UTI quantitative test. The LE-PAD consists of a mixed coating of 3-(N-tosyl-L-alaninyloxy)-5-phenylpyrrole (PE) and 1-diazo-2-naphthol-4-sulfonic acid (DAS) accumulated on an Ag film or Ag NPs. The analysis indicated that the LE amount calculated by LE-PADs was descriptive of UTI diagnosis, with an area of 0.875 (95% confidence interval, 0.704–1.000) under the receiver operating characteristic curve. Using an acceptable cut-off value, the LE-UTI PAD’s diagnosis specificity and sensitivity were 87.5% and 92.3%, respectively, while urine dipstic LE positivities were 62.5% and 76.9%, respectively. The LE-PAD displayed positive and negative probability ratios of 11.38 and 0.14 for UTI diagnosis, indicating that the new LE-PAD may be a valid method.

Novel biosensors have been recently developed based on the unusual plasmonic energy exchange of nanometallic crossed surface-relief gratings (CSRGs). CSRG-based nanosensing, nevertheless, has been restricted to spectroscopic methods and has not exploited its capacity for incorporation with pervasive electronic devices [109]. Nair et al. [109] introduced a novel nanosensor using surface plasmon resonance imaging (SPRi) allowed by CSRGs. The imaging system used two-dimensional nanoplasmonic gratings to facilitate a specific transfer of plasmonic energy between metallic nanomaterials. Finite-difference time-domain (FDTD) simulations confirmed that, due to plasmon resonance at the metal–dielectric interface, CSRG-enabled SPRi was accompanied by an electric field intensity enhancement of about 30 times. The rapid (<35 min) and label-free detection of UPEC in phosphate-buffered saline (PBS) and in human urine samples from 103 to 109 cfu/mL showed the performance of the system for biomedical applications. The platform’s LOD was around 100 cfu/mL, i.e., three orders of magnitude below the clinical LOD for UTI detection. The sensing ability of the platform was demonstrated experimentally by the diagnosis of differences in the bulk refractive index (RI), with a sensitivity of 382.2 nm/RI units (RIU) and a resolution of 10^−6^ RIU. Brayner et al. reported studies of cellular internalization of ZnO nanoparticles on *Escherichia coli* bacteria [110].

Table 1 summarizes the most commonly used NP-based sensors for the diagnosis of UTIs.

## 4. Nanomaterials for the Treatment of UTIs

Compared with conventional antimicrobials, NP-based antimicrobials are easy to fabricate and display prolonged stability, sustained drug release, and higher therapeutic efficacy. NPs that possess antimicrobial properties themselves or increase the therapeutic efficiency of antibiotics are referred nanoantibiotics [112]. Nanoantibiotics kill pathogens through multiple mechanisms including via formation of ROS, interference with energy transduction pathway, inhibition of DNA synthesis, and degradation of the microbial cell wall [113]. Although conventional antimicrobial therapies are effective in inhibiting bacteria, these therapies are ineffective to act against dormant intracellular pathogens due to poor penetration. As a consequence, several pathogens take advantage of this limitation and cause UTI recurrence. Nanoantibiotics possess the capabilities to target the intracellular reservoirs of the pathogens and thus to limit the recurrence of the diseases. Due to the promising advantages of MPs, several antimicrobial-laden NPs such as metals and metal oxides, dendrimers, surfactant-based NPs, and carbon-based nanomaterials have been employed for UTI therapy [114].

### 4.1. Metallic NPs with Antimicrobial Activity

**Figure 2:**
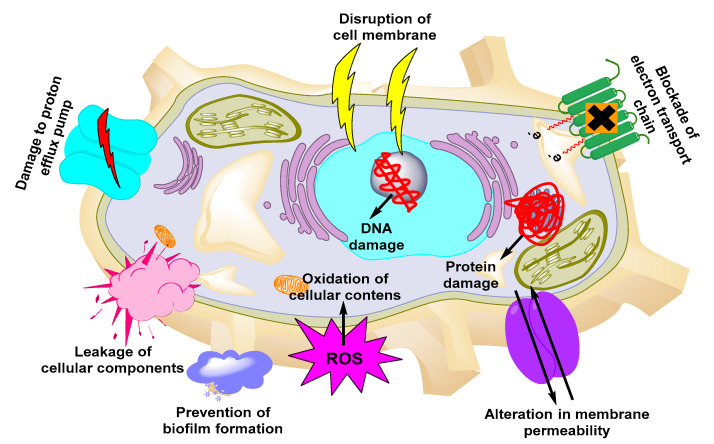
Mechanisms of action of metallic NPs against UTIs.

Due to their small size, high surface area, shape, and surface charge, metallic NPs are the most suitable nanocarriers for higher cellular uptake of antimicrobials. The conjugation of certain functional groups on their surface increases the cellular interaction of the NPs [115]. Owing to their small size, metallic NPs also exhibit higher cellular penetration than free antimicrobials and are easily absorbed from the blood circulation to the target site. Due to these unique features, several groups employed metallic NPs to achieve efficient delivery of antimicrobials against UTIs [116]. Among the several kinds of metallic NPs, the mechanism of some of the new NPs is still not understood [117,118,119,120,121,122]. The mechanisms employed by metallic NPs to exert antimicrobial activities are presented in Figure 2. Examples of metallic-based NPs with antimicrobial properties include iron-, Ag-, Au-, Ag oxide-, titanium dioxide-, aluminum oxide-, copper oxide-, and gallium-based NPs [123,124].

### 4.2. Silver-Based NPs

When the particle size of Ag is reduced to the nanorange, it exhibits antibacterial activities against *Staphylococcus* *aureus* and *Escherichia coli*. Ag NPs exhibit antimicrobial properties by affecting the division and respiratory system of microorganisms. Even though the long-term exposure to soluble Ag causes adverse effects on the intestinal tract and kidneys, Ag NPs exhibit negligible effects on human health.

Synthesis of silver-based and other metallic NPs can be achieved through physical, chemical, and biological methods. However, physical and chemical methods are associated with a number of drawbacks including the production of hazardous wastes, the difficulty in scale-up production, and the instability in aggregation while requiring expensive organometallic precursors for the synthesis of NPs. On the other hand, biological methods are regarded as safe procedures, using bacteria as potential biofactories for the synthesis of metallic NPs, making them more “eco-friendly” systems than physical and chemical methods. To exploit the advantages of eco-friendly approach, Divya et al. [125] synthesized eco-friendly Ag NPs with antimicrobial properties against several UTI pathogens and coated them on catheters that overall displayed antibiofilm and antimicrobial properties.

Saleh et al. [126] formulated and investigated the mechanisms of antimicrobial action of two different types of metallic NPs, i.e., Ag-based and titanium dioxide-based NPs, against *Proteus vulgaris* and *Proteus mirabilis*, reporting that both kinds of NPs inhibited the microbial species via downregulation of fliL gene expression directly associated with blockade of the movement of the *Proteus* species. Similarly, Das et al. [127] created Ag NPs loaded with the extract of *Oxalis corniculata* and observed that compared with pure extract, extract-based Ag NPs exhibited significantly higher antimicrobial activities against both Gram-positive and Gram-negative bacterial strains, the leading cause of UTIs. In addition, the authors also reported that the formulations allowed for significantly higher kidney stone dissolution, an effect further increased in the presence of Ag NPs.

*Pseudomonas aeruginosa* is considered a critical agent for the formation of antibiotic-resilient biofilms, reducing the therapeutic efficacy of antimicrobials. While Ag NPs are seen as an efficient choice to overcome such events due to their innate bactericidal potential, they may be toxic when used at high concentrations. To tackle such an issue, Bhargava et al. [128] functionalized the surface of the Ag NPs with fucose to increase their interaction with the lectin B component of the bacterial cell wall. The results of the study demonstrated a superior bactericidal and antibiofilm activity of the functionalized NPs. The key findings of the fluorescence study and confocal laser scanning microscopy revealed that the bactericidal activity was achieved due to the generation of higher ROS and oxidative stress-induced membrane damage. In addition, the functionalized NPs also exhibited a higher antibiofilm-forming potential through the downregulation of several virulence genes.

MDR has become a worldwide issue, showing the urgent need to develop strategies to overcome this critical problem. In this regard, Lopez-Carrizales et al. [129] developed Ag-based NPs to co-deliver amikacin and ampicillin versus single antimicrobial treatment. When ampicillin was administered in combination with Ag NPs against twelve microbial strains of UTIs, the system displayed synergistic effects against one strain, partial synergistic effects against seven strains, and additive impact against four strains. When amikacin was used in combination with Ag NPs, this second system displayed synergistic impact against three strains, partial synergistic impact against eight strains, and exhibited additive effect against another single strain. The cytotoxic effects of both combination approaches at the given concentrations were insignificant relative to the use of single drugs. Based on these results, the authors concluded that MDR may be overcome through a synergistic combined approach. Similarly, El-Batal et al. [130] investigated the potential of Ag- and boron-based NPs against MDR microbial strains. The NPs were formulated through γ-radiation-induced synthesis of Ag-boron NPs using an eco-friendly approach with polyvinylpyrrolidone as the stabilizing agent. These NPs exhibited zones of inhibition of 20 mm, 18 mm, and 16 mm and biofilm inhibition of 87%, 85.3%, and 69.4% against *Candida albicans*, *Escherichia coli*, and *Staphylococcus* *aureus*, respectively. Mishra et al. [131] formulated Ag NPs loaded with timber-yielding plant extract, having a particle size less than 100 nm. The antibacterial efficacy of the prepared formulation was evaluated against 11 MDR bacterial strains responsible for UTIs. Compared with the standard gentamicin (30 μg/mL), the prepared nanoformulation (15 μg/mL) significantly reduced the zone of inhibition from 30 to 13 mm. The toxicity of the prepared formulation was also investigated in cultured lymphocytes obtained from umbilical cord blood, showing cell death below 25%, supporting the concept of safely using these systems against MDR UTIs.

Rodríguez-Serrano et al. [132] produced Ag NPs through an eco-friendly approach using soil fungal to treat UPEC UTIs. *Escherichia coli* tend to form a biofilm which reduces the penetration and the efficacy of conventional antibiotics. The biogenic Ag NPs were formulated using metabolites excreted from the fungal strains. The prepared formulations had a minimum inhibitory concentration (MIC) of 7.5 mg/mL with a 97% reduction in biofilm formation and an 80% destruction of the matured biofilm, while the control group had a MIC value of 25 mg/mL.

Foley catheters are essential in the healthcare unit and pathogens tend to form biofilms on the surface of catheters, leading toward UTIs. To prevent this problem, researchers functionalized the surface of catheters using a combination of Ag NPs and antibiotics [133]. NPs were produced in a size range of 42–75 nm for investigation in vivo in mice. The combination of amikacin, nitrofurantoin, and Ag NPs exhibited synergistic activity. Mice treated with the combination approach did not show any kind of colonization until day 14. After 2 years, the catheters treated with antibiotics exhibited a 25% reduction in bacterial adhesion while the group treated with Ag NPs along with antibiotics displayed a 90% decline in bacteria adhesion. Shafreen et al. [134] also formulated Ag NPs using fresh water diatom, showing inhibition against *Escherichia coli* at a concentration of 300 ng/mL and decreasing biofilm formation. Confocal laser microscopy further showed that the architecture of the biofilm was also significantly decreased. Valsalam et al. [135] prepared Ag NPs through an eco-friendly approach using leaf extract from *Tropaeolum majus*. Both Ag NPs and plant extract inhibited *Pseudomonas aeroginosa* at a concentration of 6.25 μg/mL relative to other microbial strains and exhibited antifungal and anticancer activities that may be used for the treatment of other infectious diseases.

### 4.3. Copper NPs

Due to the redox-active properties of copper, several groups employed copper-based NPs for their activity against UTIs. In this regard, Al-Enizi et al. [136] formulated a copper NP-based hydrogel matrix, with a particle size distribution of 7-12 nm. The antimicrobial and cytotoxic activities of the formulation were evaluated in UTI microbes (*Escherichia coli*, *Klebsiella pnemoniae*, *Pseudomonas aeruginosa*, *Proteus vulgaris*, *Staphylococcus aureus*, *Proteus mirabilis*) and HeLa cell lines. Compared with a simple gel matrix, the copper NP-based hydrogel matrix exhibited a significantly higher zone of inhibition against UTI pathogens while cytotoxicity studies revealed the suitability of the prepared carrier system for biomedical applications. Shalom et al. [137] fabricated zinc-doped copper oxide NPs for the treatment of catheter-linked UTIs. The NPs were deposited on the surface of the catheter through the sonochemical technique. The surface-coated catheter exhibited higher antibiofilm property, biocompatibility, reduced cytokines secretion, lower cytotoxicity, and absence of irritation in rabbits. The coated catheters did not produce UTIs even 7 days after application while the uncoated catheters exhibited UTIs within 4 days.

### 4.4. Silicone Dioxide-Based NPs

Muslim et al. [138] generated tannase-conjugated silicone dioxide-based NPs through laser ablation technique, noting higher therapeutic effectiveness and significance levels against all UTI pathogenic strains compared with control groups, as an effective approach against UTIs.

### 4.5. Zinc Oxide NPs

Zinc oxide NPs exhibit cell internalization and bactericidal effects against several microbial strains involved in UTIs. Due to these properties, several researchers used zinc oxide (ZnO)-based NPs for therapy against UTIs.

In this context, Santhoshkumar et al. [139] fabricated *Passiflora caerulea* leaf extract-based ZnO NPs. Compared with simple leaf extract, the leaf extract-laden ZnO NPs exhibited a significantly higher zone of inhibition in the pathogenic microbes extracted from patients’ urine containing UTIs. Abd Elkodous et al. [140] examined the antimicrobial and antibiofilm properties of ZnO NPs against MDR bacterial strains involved in UTIs. The NPs exhibited a particle size of 69 nm, a surface area of 10.66 m^2^/g, and porosity of 13.16% and displayed antimicrobial and antibiofilm activities against all the pathogenic UTI strains. Tiwari et al. [141] investigated the potential of ZnO NPs against carbapenem-resistant microbial strains. The NPs were 30 nm in size and displayed significant antibacterial activities against the resistant strains. The authors also investigated the mechanisms of microbial inhibition and found that ZnO NPs promoted the production of ROS which, in turn, increased the peroxidation of the bacterial membrane lipids and the leakage of DNA, reducing sugars, proteins, and ultimately leading to a reduction of the cell viability. These results demonstrated that ZnO NPs have the potential to be effectively used against the resistant strains of UTIs. Similarly, El-Rab et al. [142] evaluated the inhibition potential and mechanism of ZnO NPs against the MDR strains of *Escherichia coli* and *Escherichia hermannii*. The NPs inhibited *Escherichia coli* at a concentration of 10 μg/mL and *Escherichia hermannii* at 40 μg/mL. The MIC values reported in the study were also significantly lower than in earlier evaluations. The authors also evaluated the mechanisms of inhibition of the microbes and an SEM analysis showed the distortion and blebbing of the microbial membrane, with an elongation of the cell membrane and a discharge of cellular contents. In another study, Hosseini et al. [143] tested the impact of ZnO NPs on the initiation, adhesion, agglutinin sequence, and gene expression in biofilm formation. The NPs displayed size of 20–40 nm and MIC in the range of 0.02–18.1 μg/mL. The authors observed that at this concentration, the NPs induced an initial inhibition and then tremendous reduction of the expression of genes responsible for biofilm formation. Such NPs may thus be effectively used to prevent biofilm formation with increased efficacy against microbes. Hosseini et al. [144] evaluated the impact of ZnO NPs on the adhesion of *Candida albicans* on surface catheter and biofilm formation. Among the several bacterial species tested, *Candida albicans* fluconazole-resistant strains exhibited higher adherence to the surface of catheters. The surface-adhered catheters were then treated with ZnO NPs, leading to a reduced biofilm mass. This study illustrated that ZnO NPs may be additionally used for the treatment of catheter-allied UTIs.

### 4.6. Selenium-Based NPs

El-Sayyad et al. [145] formulated gentamicin-laden selenium NPs to compare and increase the antibiofilm and antimicrobial activities of conventional gentamicin formulations against MDR microbial strains. The NPs had a particle size distribution in the range of 22–33 nm and displayed antimicrobial properties against all MDR strains of UTIs. The formulation also showed enhanced antibiofilm properties of 88% against *Staphylococcus aureus*, 87% against *Pseudomonas aeruginosa*, and 85% against *Escherichia coli*.

### 4.7. Sulfur-Based NPs

The majority of microbial strains exhibit resistance to trimethoprim and amoxicillin. To overcome such resistance, Paralikar et al. [146] prepared sulfur NPs (SNPs) loaded with trimethoprim and amoxicillin with a size in the range of 20–86 nm and with lower polydispersity index. SNPs combined with trimethoprim and amoxicillin exerted a synergistic effect and higher zone of inhibition against the resistant microbial strains compared with unloaded SNPs.

### 4.8. Polymeric NPs

**Figure 3:**
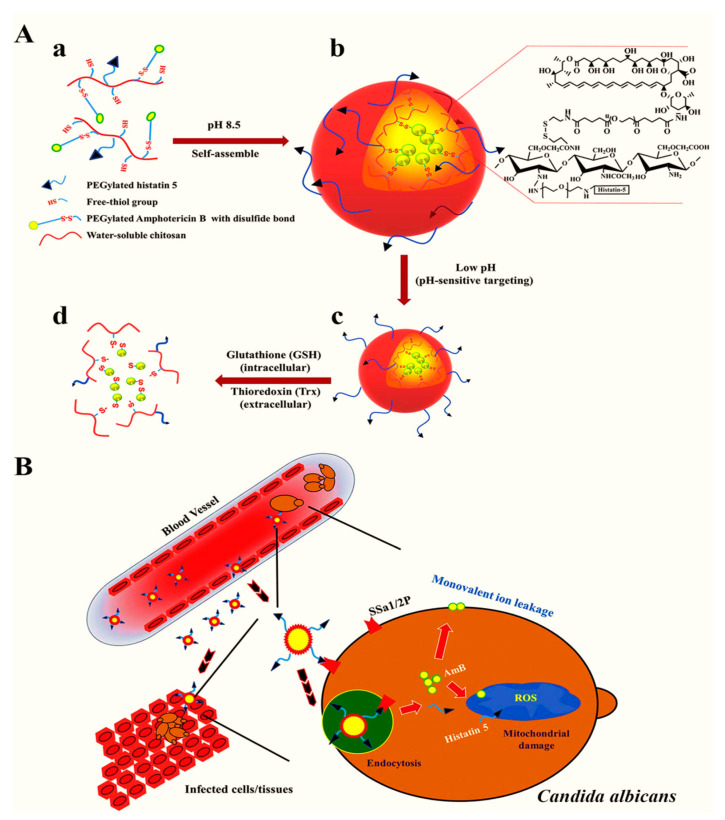
Conjugation of histatin 5 and amphotericin B with NPs. (**A**) conjugation of histatin 5 with chitosan and conjugation of a redox linker with amphotericin B (a), self-assembly of NPs through disulfide bonds between polymeric chains and formation of micelles through hydrophobic interaction of amphotericin B (b), pH-dependent activation of the targeting ligand, i.e., histatin 5 (c), release of amphotericin B through a reduction mechanism within the fungal cell (d). (**B**) Targeted uptake and intraendosomal release of amphotericin B and synergistic action of histatin B against fungal destruction. Reproduced from [147], with permission from Elsevier, 2021.

Due to their high stability, encapsulation efficiency, and ability to modify the surface properties, polymeric NPs are extensively utilized as a nanocarrier for the efficient loading of antimicrobial agents. To exploit such properties, Park et al. [147] generated a pH-sensitive and redox-responsive polymer-based nanocarrier for the efficient delivery of amphotericin B. The authors further attached the functional moieties with an antifungal histatin 5 peptide allowing for targeted delivery and displaying antifungal properties against *Candida albicans*. The mechanisms of targeted delivery are presented in Figure 3. Compared with free amphotericin B, the nanoformulation exhibited higher therapeutic efficacy and targeted delivery to the pathogenic microbe-laden cells at lower doses while reducing the cytotoxicity to healthy cells.

Crystalline deposition and formation of biofilms are the common reasons for the failure of prolonged urinary catheter-based therapy against UTIs. Bacteria colonize the surface of the catheter, block it, ultimately leading to serious UTIs. To address this problem, Dayyoub et al. [148] developed a new strategy to counteract the adhesion of pathogens to the catheter surface based on a nanoformulation consisting of norfloxacin, Ag NPs, and polylactic-co-glycolic acid (PLGA)-based polymers which underwent degradation in the aqueous environment and produced alkali products through the hydrolysis of the urea. The authors examined the adhesion of the NPs embedded with tetraether lipids on silicone and polyurethane sheets. Compared with commercial formulations, the current NPs exhibited significantly higher anti-adhesion and antimicrobial properties. An in vivo encrustation model revealed that the lipid coating reduced adhesion for 2 weeks, supporting the concept of using such a system as an efficient coating for urinary catheters.

Intravesical therapy used for therapy against bladder cancer may be further adapted for the treatment of UTIs, although certain issues associated with this technique still need attention such as a short dwelling and washout period. In this regard, Brauner et al. [149] formulated two types of PLGA 2300- and PLGA 503H-based NP surface tailored with wheat germ agglutinin. Both types of NPs significantly enhanced the adhesion of the NPs to human uroepithelial cells while the loading of trimethoprim did not alter the adhesion potential of the NPs. The highest adhesion was observed in sodium bicarbonate buffer at pH 5. The dwelling time was in the range of 15–30 min, and after 15 min less than 50% of the NPs were bound to the surface while more than 70% were bound after 30 min, without further increment beyond this time point. Such surface-functionalized NPs may thus be used as a promising approach for therapy against UTIs.

Amphotericin B is a commonly used antibiotic for UTIs, yet it may lead to hemolytic anemia and nephrotoxicity. In an attempt to reduce such hurdles, Ludwig et al. [150] created PLGA-based NPs decorated with chitosan for the efficient loading of amphotericin B. The NPs exhibited a size of 460 nm and encapsulation efficiency of 46%. Compared with free drugs, amphotericin B-loaded NPs exhibited lower MIC, a higher zone of inhibition, and reduced adverse effects.

Sharma et al. [151] developed chitosan-based zingerone NPs against *Pseudomonas aeruginosa* to treat catheter-induced UTIs. Due to the antimicrobial properties of chitosan, these NPs exhibited a synergistic effect against the pathogens without toxicity in kidney cell lines. Findings in vivo revealed that chitosan-based NPs decreased the bacterial cell count in the bladder and renal tissues and ex vivo studies evidence the intracellular killing of the pathogen and higher uptake by phagocytes with an important reduction of the level of several inflammatory mediators.

### 4.9. Hybrid NPs (Polymeric/Metallic NPs)

One of the reasons for the occurrence of UTIs is the presence of the ureteral stent and the pain associated with extraction. In this regard, Gao et al. [152] prepared an extraction of free, biodegradable, and antibacterial stent having a continuous renewable surface and antibiofilm properties through the construction of poly(amide-amine)-capped Ag shell and Au core nanoparticle (Ag@Au NP)-embedded fiber membrane-structured poly(glycolic acid) (PGA)/PGLA NPs. The stent exhibited a rapid killing kinetics of *Staphylococcus aureus* and *Escherichia coli* within 10 and 5 min, respectively, along with an inhibition rate of up to 99%. The gradual degradation of PGA and PLGA self-cleaned the stent and also displayed antibacterial properties through constant exfoliation of the surface of the stent. The stent also exhibited biocompatibility, lower cytotoxicity, and bactericidal activity. In vivo studies in pigs revealed that the stent had significant antibiofilm properties and decreased the levels of necrotic and inflammatory cells, as a promising and extraction-free approach to treat UTIs.

Ashmore et al. [111] compared the antimicrobial properties of polymer-coated Ag NPs, i.e., 10% Ag (Ag 10% + polymer) and 99% Ag (Ag-polyvinylpyrrolidone—AgPVP), versus uncoated NPs. NPs coated with polymer exhibited higher potency against *Escherichia coli* compared with the uncoated NPs, with bacterial growth inhibited within 8 h at a concentration of 0.156 mg/mL while uncoated NPs were effective at 0.312 mg/mL. An SEM analysis revealed that the formulations destroyed the cell membrane, leading to bacterial cell rupture and expulsion of the cellular content.

*Acinetobacter baumannii* is one of the several microbes with MDR against UTIs, with all conventional antibiotics being largely ineffective to prevent their occurrence. Plant-based extracts have currently gained much attention to avoid the problem of MDR. With this in mind, Tiwari et al. [153] investigated plant extracts from various species to explore their antimicrobial activities against MDR pathogenic microbes. Among them, a complete extract or primary and secondary metabolites from *Phyllanthus emblica* showed the highest inhibition potential for carbapenem-resistant *Acinetobacter baumannii* via the production of ROS, carbonylation of proteins, and membrane damage. To further confirm the antimicrobial properties of the secondary metabolites, gallate (the major secondary metabolite of the plant)-laden PVP-capped hybrid Ag NPs (G-PVP-Ag NPs) were formulated and had significant antimicrobial activity also via ROS production.

### 4.10. Surfactant-Based NPs

*Pseudomonas aeruginosa* is the microorganism that causes hospital-acquired UTIs. This organism can grow in a free form as well as on biofilm colonies attached to a surface. When the species grows in the biofilms, the presence of biofilms limits the efficacy of conventional formulations, leading to its resistance. To overcome this issue, Lopes et al. [154] evaluated the anti-biofilm potential of a glycerol monolaurate (GML) nanoformulation with an average diameter of 190 nm. Compared with free GML, GML nanocapsules reduced the MIC from 62.5 to 15.62 μg/mL as well as the biofilm mass, polysaccharides, proteins, and the presence of *Pseudomonas aeruginosa* on the biofilms.

### 4.11. Carbon-Based Nanomaterials

Carbon-based nanomaterials have been widely employed due to their unique optical and tunable surface properties, low systemic toxicities, cost-effectiveness, and antimicrobial activities. Due to these features, Lu et al. [155] took advantage of carbon-based materials against UTIs, such as graphene, carbon nanotubes, and nanodiamonds. Dybowska-Sarapuk et al. [156] also formulated carbon-based nanolayers using graphene decorated with Ag NPs against the formation of *Staphylococcus epidermidis*-induced biofilm on the surface of the Foley catheter. Such graphene-nanoAg nanolayers were capable of preventing biofilm formation on the surface of the catheter, as a strong tool against the complications associated with UTIs. Rouhani et al. [157] functionalized amoxicillin-based nanodiamonds with polyethyleneimine (PEI) and conjugation with ferromagnetic material (Fe_3_O_4_) to achieve active targeted delivery. The nanodiamonds were capable to release the drug in the target sites to overcome the problem of dose-related resistance associated with a free drug approach against UTIs.

It is estimated that there are 25–45% chances of relapse in women suffering from UTIs mostly due to *Escherichia coli* infection, as a result of the internalization of the pathogen in the urinary bladder and since antibiotics fail to reach the target site. To tackle this problem, Iyer et al. [158] prepared two types of nanodiamonds with sizes of 6 and 25 nm to kill intracellular and extracellular pathogens. Nanodiamonds with a size of 6 nm had a more potent internalization capability than 25 nm-sized systems, using a mechanism based on actin-dependent endocytosis, and better reduced bacterial infiltration, improving the killing kinetics of the *Escherichia coli*.

### 4.12. Dendrimer-Based Nanomaterials

Dendrimers are artificial macromolecules with several functional groups and an intact molecular structure, playing an increasing role in drug delivery via the fabrication of nanoscale particles. Sehad et al. [159] created surface-functionalized dendrimers by attaching mannose sugar on the surface of the dendrimers through copper-catalyzed cyclo-addition click reaction. The mannosylated dendrimers were constructed to examine the impact of mannosylation upon the binding capacity of dendrimers on the bacterial cell surface. The dendrimers had the ability to bind with FimH type-1 lectins present at the tip of bacterial fimbriae, showing high potency, bioadhesion, and antibiofilm properties against *Escherichia coli*. Zhu et al. [160] generated mannoside-decorated dendrimers to increase the formation of a protecting coating to avoid the adhesion of pathogenic microbes at the surface of silicone catheters. The authors reported that 95% of nonpathogenic microbes were retained on the catheters while all MDR pathogenic strains were excluded from colonization at their surface. The most commonly used nanoparticle-based delivery approaches against several species of UTIs are presented in Table 2.

## 5. Conclusions, Challenges, and Perspectives

Compared with conventional therapies, NP-mediated delivery of antimicrobials displays higher therapeutic efficacy by minimizing off-target delivery and by managing MDR. The issue of biofilm formation, an additional barrier that impairs the therapeutic efficacy of antimicrobials, may also be addressed using the nanotechnology procedure, especially when using metallic NPs that possess an intrinsic antibiofilm formation potential. With the emerging role of nanotechnology in drug delivery and diagnostics, this approach may further overcome the limitations of conventional therapies in clinical trials, making it the most appealing strategy for the diagnosis, treatment, and prevention of UTIs. In order to minimize the seriousness of these infections, the early diagnosis of UTIs will play a major role in their rapid eradication, and any delay in effective UTI detection may result in the development of intracellular reservoirs particularly difficult to eliminate. The integration of nanomaterials with the currently available diagnosis methods will be a strong option for the early-stage identification of UTIs and may solve the major constraints in evaluation and therapy, including infection recurrence. Although NPs have the potential to treat and diagnose UTIs, there are still several challenges that need to be addressed prior to their successful translation in the clinics including the investigation of the interaction between NPs and cells, tissues, and organs, the identification of the most appropriate routes of administration, and most importantly the evaluation of toxic responses to long-term exposure to NPs. Despite several advancements regarding the value of NPs in the diagnosis and treatment of UTIs, it will be important to examine the pathways through which NPs exert their therapeutic efficacy. There is also limited information on the metabolism and clearance of NPs and on the nature of their targets. The specific combination of NPs and antimicrobials may reduce the emergence of multidrug resistance of bacteria to drug sensitivity and translation to clinical practice will require a thorough investigation of the pharmacokinetic and pharmacodynamic profiles of these NPs. Finally, for innovative research, the limits of pathogen detection and the time of the proposed analysis will have to be significantly improved.

## Figures and Tables

**Table 1:** Most commonly used nanoparticle (NP)-based sensors for the diagnosis of UTIs.

Nanosensors	Application	References
bacteria-grabbing nanochip-based on SERS	detection of *Proteus mirabilis* PRM1, *Escherichia coli* CFT 073, and *Pseudomonas aeruginosa* PAO1 uropathogens	[99]
nanopaper-based systems	detection of LE (LE-PAD) as a proof-of-concept for UTI quantitative testing	[108]
MNPs identification of	*Pseudomonas aeruginosa* in vivo	[111]

Abbreviations: SERS, surface-enhanced Raman scattering; MNPs, magnetic nanoparticles; LE, leukocyte esterase; LE-PAD, leukocyte esterase-paper-based analytical device; UTI, urinary tract infection.

**Table 2:** Most commonly used NP-based delivery approaches for therapy against UTIs.

NPs		Pathogens	Properties	References
metallic NPs	Ag	*Escherichia coli*	MIC of 7.5 mg/mL, 97% reduction of biofilm formation, 80% destruction of matured biofilms	[132]
	Copper	*Escherichia coli*, *Klebsiella pnemoniae*, *Pseudomonas aeruginosa*, *Proteus vulgaris*, *Staphylococcus aureus*, *Proteus mirabilis*	high zone of inhibition against UTI pathogens, low cytotoxicity of the NPs	[161]
	ZnO	*Escherichia coli*, *Escherichia hermannii*	anti-bacterial effects at 10 and 40 μg/mL (*Escherichia coli*, *Escherichia hermannii*), low MIC	[162]
polymeric NPs (chitosan-based NPs)		*Candida albicans*	high therapeutic efficacy and targeting of pathogenic microbe-laden cells, low cytotoxicity	[163]
hybrid NPs		*Escherichia coli*	high potency (inhibition of bacterial growth within 8 h at 0.156 mg/mL)	[111]
carbon-based NPs		*Staphylococcus epidermidis*	prevention of biofilm formation on Foley catheter by graphene-nano Ag nanolayers	[164]

Abbreviations: NPs, nanoparticles; Ag, silver; ZnO, zinc oxide; MIC, minimum inhibitory concentration; UTI, urinary tract infection.

## Data Availability

Not applicable.
